# Can Psychological, Social and Demographical Factors Predict Clinical Characteristics Symptomatology of Bipolar Affective Disorder and Schizophrenia?

**DOI:** 10.1007/s11126-015-9405-z

**Published:** 2015-12-08

**Authors:** Malgorzata Maciukiewicz, Joanna Pawlak, Pawel Kapelski, Magdalena Łabędzka, Maria Skibinska, Dorota Zaremba, Anna Leszczynska-Rodziewicz, Monika Dmitrzak-Weglarz, Joanna Hauser

**Affiliations:** Laboratory of Psychiatric Genetics, Department of Psychiatry, Poznan University of Medical Sciences, Rokietnicka St. 8, 60-806 Poznan, Poland; Pharmacogenetics Research Clinic, Campbell Family Mental Health Research Institute, Centre for Addiction and Mental Health, Toronto, ON Canada; Department of Psychiatry, University of Toronto, Toronto, ON Canada

**Keywords:** Schizophrenia, Bipolar affective disorder, OPCRIT, Dimensions

## Abstract

Schizophrenia (SCH) is a complex, psychiatric disorder affecting 1 % of population. Its clinical phenotype is heterogeneous with delusions, hallucinations, depression, disorganized behaviour and negative symptoms. Bipolar affective disorder (BD) refers to periodic changes in mood and activity from depression to mania. It affects 0.5–1.5 % of population. Two types of disorder (type I and type II) are distinguished by severity of mania episodes. In our analysis, we aimed to check if clinical and demographical characteristics of the sample are predictors of symptom dimensions occurrence in BD and SCH cases. We included total sample of 443 bipolar and 439 schizophrenia patients. Diagnosis was based on DSM-IV criteria using Structured Clinical Interview for DSM-IV. We applied regression models to analyse associations between clinical and demographical traits from OPCRIT and symptom dimensions. We used previously computed dimensions of schizophrenia and bipolar affective disorder as quantitative traits for regression models. Male gender seemed protective factor for depression dimension in schizophrenia and bipolar disorder sample. Presence of definite psychosocial stressor prior disease seemed risk factor for depressive and suicidal domain in BD and SCH. OPCRIT items describing premorbid functioning seemed related with depression, positive and disorganised dimensions in schizophrenia and psychotic in BD. We proved clinical and demographical characteristics of the sample are predictors of symptom dimensions of schizophrenia and bipolar disorder. We also saw relation between clinical dimensions and course of disorder and impairment during disorder.

## Background

Schizophrenia (SCH) is a complex, psychiatric disorder with a mean lifetime morbid risk 1 % [1]. Its clinical phenotype is heterogeneous with delusions, hallucinations, depression, bizarre or disorganized behaviour and negative symptoms. Depressive episodes are also observed during SCH course [2]. Schizophrenia is influenced by both genetic and environmental factors [3]. Its exact etiology is still undescribed, thus Riley [4] suggested it is rather genetically mediated than genetically determined (H = 0.8). There are several environmental risk factors of schizophrenia, including: premature birth and low birth weight [5], maternal infections during pregnancy [6], hypoxia during neurodevelopment [7], seasonality of birth [8]. There are also psychological risk factors, including family instability and trauma during childhood [9].

Bipolar affective disorder (BD) refers to periodic changes in mood and activity from depression to mania. It affects 1 % of population. Two types of disorder (type I and type II) are distinguished by severity of mania episodes [10]. Mixed states are also observed. Psychotic symptoms are observed in some cases [11]. Family history of bipolar disorder is important risk factor [12]. As in schizophrenia, environmental risk factors of disorder are known. Dysfunctional interactions among family members increase the risk [13].

Schizophrenia and BD are both complex in terms of both clinical and genetic picture. Thus many factor analysis studies aimed to find clinical dimensions. Depressive, positive, negative, excitement and disorganised domains were detected in SCH sample, whereas depressive, excitement and psychotic appeared for BD [14–18]. Potentially useful strategy is to use previously computed factor structure to seek for association with pre-morbid risk factors [15].

Both disorders (SCH and BD) are characterised by substantial genetic overlap. The genetic correlation coefficient equalled 0.6. Results from twin and adoption studies suggests overlap with schizoaffective disorder as well [19]. The large clinical overlap between schizophrenia and bipolar affective disorder is also known [20, 21].

In our study, we investigated if clinical and demographical characteristics of the sample (e.g. age at onset, duration of illness, sex) are predictors of symptom dimensions. Clinical dimensions describe disorder diversity and severity. We analysed if/how socio-demographical and clinical characteristics influence symptomatology of SCH and BD. To achieve our goal, we applied previously computed factor structure [14] as quantitative trait for regression models.

## Sample Analysed

The sample comprised 892 bipolar disorder (n = 443) and schizophrenia (n = 449) patients. Diagnosis was based on DSM-IV criteria using Structured Clinical Interview for DSM-IV (SCID) [22]. We collected data about familial burden of the psychiatric disorders where possible. Lifetime perspective of symptoms was based on OPCRIT [23] checklist.

The average age at onset of BD individuals was 30.62 (SD = 11.17), whereas in schizophrenia cases 23.4 (SD = 6.59). Longer duration of illness appeared for SCH patients (22.25; SD = 21) in comparison with BD ones (19.37; SD = 14.83). Majority of patients were employed before onset of disorder (53 % in SCH and 83 % in BD).

All subjects were inpatients from Wielkopolska region of Poland. Patients gave written consent for the study after being informed about its details. Local Bioethics Committee approved the study. Sample characteristics is depicted in the Table 1.

**Table 1:** Sample characteristics. NA’s (“not available”) refers to situation when there are absences in OPCRIT items

	Schizophrenia (n = 439)	Bipolar affective disorder (n = 443)
Sex	Female: 224 (51 %)	Female: 252 (57 %)
	Male: 215 (49 %)	Male: 191 (43 %)
Age at onset	Mean: 23.08	Mean: 30.62
	Min: 5.0	Min: 10.0
	Max: 52.0	Max: 63.0
	SD: 6.59	SD: 11.17
Family history of schizophrenia	Absent: 346 (79 %)	Absent: 409 (93 %)
	Present: 81 (18 %)	Present: 24 (5 %)
	NAs: 12 (3 %)	NAs: 10 (2 %)
Family history of other psychiatric disorders	Absent: 307 (70 %)	Absent: 216 (49 %)
	Present: 108 (25 %)	Present: 221 (50 %)
	NAs: 12 (5 %)	NAs: 6 (1 %)
Marital status	Married: 107 (24 %)	Maried: 307 (69 %)
	Single: 330 (75.8 %)	Single: 135 (30 %)
	NAs: 2 (0.2 %)	NAs: 1 (1 %)
Employment status at onsent	Employed: 228 (52 %)	Employed: 373 (84 %)
	Unemployed: 198 (45 %)	Unemployed: 63 (14 %)
	NAs: 13 (3 %)	NAs: 7 (2 %)
Definite psychosocial stressor prior to onset	Absent: 338 (77 %)	Absent: 243 (55 %)
	Present: 89 (20 %)	Present: 162 (36 %)
	NAs: 12 (3 %)	NAs: 33 (9 %)
Average duration of episode in weeks	Mean: 20.99	Mean: 19.37
	Min: 1	Min: 2
	Max: 240.00	Max: 156.00
	SD: 21	SD: 14.83

## Statistical Analysis

We applied Poisson regression models to detect relations between symptom dimensions and social/demographic characteristics. Previously described dimensions worked as quantitative dependent variable [14], thus we used Poisson instead of logistic regression. We defined quantitative trait (symptom dimension) as a sum of appropriate OPCRIT ratings. Item composition of particular domains is shown in Table 2. The high score of dimension (for example depressive) means, that most items generating domain ratings exceeding 0. It is not identical with severe depression identified by clinical terms.

**Table 2:** Previously described dimensional structure of schizophrenia and bipolar affective disorder [14]

	Dimension	OPCRIT items
Schizophrenia	Depression (main)	Slowed activity, loss of energy/tiredness, dysphoria, loss of pleasure, altered libido, suicidal ideation
	Appetite disturbances	Poor appetite, weight loss
	Suicidal	Excessive self reproach, delusions of guilt, nihilistic delusions
	Excitment	Excessive activity, reckless activity, distractibility, reduced need for sleep, agitated activity, pressured speech, thoughts racing, elevated mood, increased sociability
	Atypical depression	Increased appetite, weight gain
	Disorganised	Speech difficult to understand, incoherent, positive formal thought disorder, inappropriate affect
	Negative	Restricted affect, blunted affect
	Psychotic	Relationship between psychotic and affective symptoms, widespread delusions, primary delusions perception, other primary delusions
	Positive (first rank symptoms) 1	Delusions of influence, delusions of passivity, thought insertion, thought withdrawal, thought broadcast, thought echo
	Positive (first rank symptoms) 2	Delusions and hallucinations last for 1 week, persecutory/jealous delusions and hallucinations, third person auditory hallucinations, running commentary voices, abusive/accusatory/persecutory voices
Bipolar affective disorder	Depression (main)	Slowed activity, loss of energy/tiredness, dysphoria, diurnal variation, loss of pleasure, altered libido, poor concentration, excessive self reproach, suicidal ideation
	Appetite disturbances	Poor appetite, weight loss
	Atypical depression	Increased appetite, weight gain, excessive sleep
	Sleep disturbances	Middle insomnia (broken sleep), early morning waking, poor appetite
	Psychotic	Relationship between psychotic and affective symptoms, persecutory delusions, grandiose delusions, widespread delusions
	Excitement	Excessive activity, reckless activity, distractibility, reduced need for sleep, pressured speech, thoughts racing, elevated mood

For BD, we built five models, basing on information collected: (1) affective disorders in a family, bipolar disorder in the family and other disorders in a family (first and second degree relatives); (2) premorbid personality disorder, marital status, employment at onset, work adjustment, premorbid social adjustment and definite psychological stressor prior onset; (3) age at onset: early (childhood and adolescence) and late (adulthood) and sex. Similar to Goldstein, we treated onset when 19 and more as adulthood and called it late [24]. In case of schizophrenia sample we checked following models: (1) gender and age at onset. Schizophrenia, as other neuropsychiatric illnesses, starts typically in late adolescence [25]. Thus, we treated age at onset 18 and earlier as early and onset later than 19 as late; (2) premorbid personality disorder, marital status, employment at onset, poor work adjustment, premorbid social adjustment and definite psychological stressor prior onset; (3) family history of schizophrenia, family history of other psychiatric disorders.

At the final stage, we used clinical dimension to predict course and impairment/incapacity during illness. Course of disorder and impairment/incapacity during disorder are measured by OPCRIT variables. Course (scored 1–5) is described as: single episode with good recovery (1); multiple episodes with good recovery (2); multiple episodes with partial recovery (3); continuous chronic illness (4); continuous chronic illness with deterioration (5). When impairment/incapacity is measured (scored 0–3) it is reported as: no impairment (0); subjective impairment at work, school, or in social functioning (1); impairment in major life role with definite reduction in productivity and/or criticism has been received (2); no function at all in major life role for more than 2 days, or in patient treatment has been required or active psychotic symptoms such as delusions or hallucinations have occurred (3).

All computations were performed using R environment [26].

## Results

### Regression Models of Bipolar Sample

We got statistically significant models for depression and psychotic domains. Results are presented in Table 3.

**Table 3:** Prediction of dimension severity based on social and demographic data

Item	Symptom dimension	Schizophrenia			Bipolar disorder		
		OR	CI (2.5 %;97.5 %)	*p*	OR	CI (2.5 %;97.5 %)	*p*
Sex male	Main depression	0.922	0.863;0.985	0.016	0.931	0.888;0.977	0.004
	Psychotic				1.529	1.331;1.755	1.76E-009
Late age at onset	Main depression	0.745	0.692;0.802	4.92E-15			
	Psychotic				0.78	0.659;0.930	0.005
Premorbid personality disorder present	Main depression	1.275	1.143;1.422	0.0006	NS	NS	NS
	Excitement	0.535	0.373;0.768	0.001			
	Suicidal	1.455	1.070;1.979	0.017			
	Positive 1	0.645	0.493;0.844	0.001			
	Positive 2	0.703	0.557;0.888	0.003			
Definite psychosocial stressor prior to onset present	Excitement	1.360	1.147;1.613	0.0004			
	Suicidal	1.490	1.199;1.853	0.0003			
	Main depression	1.106	1.019;1.199	0.015	1.072	1.0197;1.127	0.006
Marital status single	Main depression	1.094	1.004;1.193	0.04			
	Psychotic				1.254	1.072;1.467	0.0047
Unemployed at onset	Positive 1	1.238	1.081;1.419	0.002			
	Psychotic				1.375	1.140;1.658	0.0008
Poor premorbid social adjustment no	Main depression	0.904	0.842;0.972	0.006	NS	NS	NS
	Disorganised	0.839	0.735;0.958	0.0097			
Family history of other psychiatric disorder present	*Excitement*	0.811	0.674;0.975	0.025	NS	NS	NS
	Main depression	1.174	1.090;1.264	2.28E-05			

NS abbreviates that no significant *p* value appeared for given variable

For depression domain, significant results appeared for models: (1) gender + age at onset; (2) premorbid personality disorder + marital status + employment at onset + work adjustment, premorbid social adjustment + definite psychological stressor prior onset. Male gender seemed protective factor for depression dimension (*p* = 0.004; OR 0.931). Presence of psychosocial stressor prior disease onset appeared as risk factor (*p* = 0.006; OR 1.072) for depression scores.

The same models (1) and (2) gave statistically important results for psychotic dimension. Late age at onset decreases its scores(*p* = 0.005; OR 0.783). Being unemployed before disease onset (*p* = 0.0008; OR 1.375) and having marital status “single” (*p* = 0.0047; OR 1.254) were risk factors.

### Regression Models of Schizophrenia

For schizophrenia sample, we got statistically significant models for depression, positive, disorganised and excitement dimensions (see Table 2). We got strongest results for two models: (1) gender + age at onset and (2) premorbid personality disorder + marital status + employment at onset + work adjustment, premorbid social adjustment + definite psychological stressor prior onset.

Male gender (*p* = 0.016; OR 0.922) and late age at onset (*p* = 0.0; OR 0.745) seemed protective towards depressive dimension. Presence of premorbid personality disorder (*p* = 0.0006; OR 1.275) is a risk factor for higher depression scores. Absence of poor premorbid social adjustment decreases main depressive (*p* = 0.006; OR 0.904) and disorganization (*p* = 0.0097; OR 0.839) domains scores. Presence of psychiatric disorders in family seemed risk factor for depressive dimension (*p* = 2.28 × 10^−5^; OR 1.174). “Marital status” equalled “single” increased depression scores (*p* = 0.04; OR 1.094). We detected presence of psychological stressor prior to disorder onset (*p* = 0.003; OR 1.490) and premorbid personality disorder (*p* = 0.017; OR 1.455) as increasing suicidal dimension scores.

Presence of premorbid personality disorder diminishes excitement dimension scores (*p* = 0.001; OR 0.535). When premorbid psychological stressor prior to disease onset appeared, it increases excitement domain scores (*p* = 0.002, OR 1.023). Family history of other psychiatric disorder seemed protective towards schizophrenia’s excitement dimension scores (*p* = 0.025, OR 0.811).

Two positive psychotic subdimensions (described as positive 1 and positive 2) were related to premorbid functioning traits, i.e. presence of premorbid personality disorder and being unemployed at disease onset. Being unemployed seemed risk factor (*p* = 0.002, OR 1.238) of positive 1 subdimension. Presence of premorbid personality disorder was protective towards both positive 1 (*p* = 0.001, OR 0.645) and positive 2 dimension (*p* = 0.003; OR 0.703) domains.

### Clinical Dimensions as Course Predictors

We observed relation of main depression dimension of bipolar disorder when use clinical dimensions as independent variables for regression equation. In the schizophrenia sample disorganised and excitement domains seemed important predictors of “impairment/incapacity during disorder” and “course of disorder” defined by OPCRIT checklist (see Table 4).

**Table 4:** Dimensions and prediction of disorder course and impairment/incapacity during disorders

Item	Schizophrenia			Bipolar disorder		
	OR	CI (2.5 %;97.5 %)	*p*	OR	CI (2.5 %;97.5 %)	*p*
Course of disorder	NS	NS	NS	**1.023**	**1.009;1.039**	**0.002**
Impairment incapacity during disorder	0.970;1.045	*0.948;0.993;* 1.003;1.088	*0.010;* 0.034	NS	NS	NS

NS abbreviates that no significant model obtained for given variable

Normal values indicated main depression symptom dimension; Bold values indicated suicidal symptom dimension; Italicised values indicated excitement symptom dimension; Underlined values indicated disorganised symptom dimension

Main depression domain seemed to increase risk of worse course of bipolar disorder (*p* = 0.02, OR 1.023). Course of disorder is defined by OPCRIT variables and coded numerically. “Worse course” means higher ratings in “course of disorder” item. In the schizophrenia sample, disorganization dimension was related with individual incapacity during disorder. When more disorganization symptoms appeared (higher score of domain), case’s incapacity increases (*p* = 0.034, OR 1.045). Excitement symptoms decrease (*p* = 0.01, OR 0.970) impairment and incapacity during disorder scores.

## Discussion

Previous researchers proved relations between clinical and demographical traits and psychiatric disorders. In our study we use previously computed clinical dimensions of schizophrenia and bipolar disorder to seek for its relation with sample characteristics. We applied regression to detect and estimate relations among clinical dimension and clinical and demographical characteristics. We took no assumptions before conducting analyses. Our results are consistent with previous reports about relation of clinical and demographical characteristics and symptom dimensions of schizophrenia and bipolar disorder Marital status “single”, presence of family history of psychiatric disorders and premorbid personality disorders as risk factors for depressive domains higher scores. For suicidal subdimension presence of premorbid personality disorder and definite psychological stressor prior onset increase its scores. Male gender and later age at onset seemed protective towards higher depression scores in both schizophrenia and bipolar disorder. Higher depression scores in BD increase course of disorder scores. The relations between excitement domain and family history of psychiatric disorder, premorbid personality disorders and impairment/incapacity during disorder need further investigations.

Male gender seemed protective factor towards depression dimension high scores in schizophrenia and bipolar disorder samples. The observation is consistent with the fact that depression appears more often in woman and females are more likely to have depressive symptoms than males [27]. Other research groups confirmed female sex as significant predictor of depressive symptoms [28] and definite depression [29, 30]. Study by Rodgers group showed several depression subtypes appearance varies between males and females: anxiety disorders appeared in females with typical subtype, whereas males with severe typical type exhibited less masculine orientation. Severe atypical type associated with alcohol/drug dependence in female sample only [31].

For schizophrenia individuals age at onset as important predictor for depression dimension. Late age at onset seemed protective. Study by Faravelli group showed that risk of depression increase with age, but for females before menopause only [32]. Our results might differ, because we analysed not depressive disorder, but depression dimension in a schizophrenia. Recent study by Yasuda group proved late onset of SCH is characterised by more depressive symptoms [33]. Study by Emsley showed PANSS scores for depression and anxiety symptoms are more severe in females, first-episode individuals and ones with positive symptoms predominant. Depressive/anxiety scores on the other hand correlated with age, positive scores in PANSS and treatment outcome. Researchers also suggested depressive and anxiety scores presence may predict more favourable treatment outcome [34]. In our models “late age at onset” simply means “adult onset”, whereas “early age at onset” equals “adolescent age at onset”, which may be the reasons of some dissimilarities between our results and those obtained by other research groups. Our models suggest onset after adolescence is somehow protective towards depression symptoms. Further investigations in more detailed age groups might give more insight.

We found presence/absence of premorbid personality disorder as important predictor factor for depression, positive and excitement dimensions in schizophrenia. Premorbid personality disorder seemed to reduce scores of excitement and positive domains. We called our dimension excitement not (hypo)manic as its composed from items describing symptoms (excessive activity, reckless activity, distractibility, reduced need for sleep, agitated activity, pressured speech, thoughts racing, elevated mood and increase sociability). Possible explanation is presence of variables: “increase sociability” and “excessive activity” in excitement domain. Particular premorbid personality traits were related with specific dimensions: sociopathic traits with disorganization, schizotypal with positive dimension, whereas schizoid with negative dimensions and lesser with positive one. Manic traits were associated with disorganization dimension, negative dimension with schizoid, passive-dependent and schizotypic traits [35, 36]. As Peralta previously suggested, relation between premorbid personality disorders and schizophrenia dimensions should be interpreted in caution. It is often observed than premorbid disorders are diagnosed in retrospective [37]. We interpret our results carefully. Without more in depth analyses of premorbid disorders it is difficult to explain its relation with excitement and positive domains.

Presence of premorbid personality disorders and definite psychological stressor prior onset seemed risk factor of suicidal disturbances higher scores in schizophrenia cases. Suicidal behaviour is observed during schizophrenia and associated with depression. Suicide is important cause of death in schizophrenia: 10 % [38] or as suggested by others 4–5 % [39, 40] of schizophrenia patients commit suicide. Hopelessness, depression and greater insight into illness make important risk factor for suicidal behaviour, whereas being unmarried and male gender are associated with lower suicide risk [41–43]. Clinical and demographical characteristics and known as risk or protective factors for suicide attempts. Early age at onset, poor premorbid social adjustment and childlessness in females were suggested as associated with suicide attempts in schizophrenia and affective disorders cases. Authors also stated demographical and clinical risk factors cannot be ignored [44]. Other studies however, showed that marital status, age and education do not influence suicidal ideation [45]. Good premorbid functioning as a single factor did not seem protective [46]. We described relation between presence of premorbid personality disorder and definite psychological stressor prior to disease with depressive symptoms associated with suicidal ideation (excessive self-reproach, delusions of guilt and nihilistic delusions), not suicidal behaviour. Probably interaction analyses of premorbid social adjustment and family burden of suicide attempts might produce more in depth models.

We saw relation between premorbid personality disorders and depression domain. Important aspect of depression in schizophrenia, is possible difficulty to distinguish from negative symptoms [47], which are typically present [48]. Premorbid personal disorders, depressive and negative manifestations might exhibit similar clinical picture. Apathy and lack of emotion are similar in both depressive and negative manifestations [49]. Depressive symptoms may be present in chronic phase and acute schizophrenia episode [34]. The fact that OPCRIT items describing premorbid social functioning increases severity of depressive manifestations only, might be results of difficulty mentioned. Early age at onset is associated with more severe negative symptoms of schizophrenia as [50] suggested. We saw late age at onset being protective towards depressive symptoms. However depressive and negative symptoms partly overlap in schizophrenia [51].

In schizophrenia sample, we observed presence of family history of psychiatric disorders, increases depressive dimension scores and decreases excitement dimension scores. It was described as risk of schizophrenia development [52]. We detected it is especially for depression dimension. Depressive symptoms are common in schizophrenia [51] and appear more frequent when there is family history of depression [53]. In our models “late” age at onset seemed protective towards depressive dimension in schizophrenia. No relation of family history and symptom dimensions appeared for bipolar sample.

In the last stage we checked if/how symptomatology of disease might be useful to predict disorder course and social functioning (described by OPCRIT variables “impairment/incapacity during disorder” and “course of disorder”). Higher scores of depression domain are associated with higher scores in course of disorder in BD sample. In case of schizophrenia, “disorganised” dimension seemed risk factor for worse impairment/incapacity during disorder. Excitement domain in SCH decrease impairment/incapacity, however this result are to interpret carefully. Correlation between depressive symptoms and performance and interpersonal behaviour was described. Mania symptoms seemed related with interpersonal friction [54]. Our bipolar models suggests association between high depression scores and high scores in course of disorder. Higher scores in course of disorder correspond to worse remission Partial remission with residual symptoms often happen in bipolar and unipolar disorder [55, 56]. In bipolar sample subsyndromal residual symptoms are related to last episode [57]. In the schizophrenia cases, “disorganised” dimension lead to poor social functioning during its course. Excitement dimension, however seemed protective. The reason might be excitement/mania domain definition. “Increased sociability”, “excessive activity” variables might work protective towards social isolation. Disorganization often present in schizophrenia has negative impact of social functioning. Recent study by Pandina proved clinically positive changes in disorganization symptoms enhances individual’s overall functioning [58].

Statistical methods enable researchers to detect and describe relationships. Pandina group applied regression models to analyse clinical symptoms and demographic characteristics influences cognitive improvement in schizophrenia and schizoaffective disorder [58]. Fiedorowicz group proved family history of bipolar disorders influence illness course, by increasing risk of hypomania/mania episodes frequency [59]. Recently Skokou and Gourzis checked how age at onset, sex, habitat, marital status and premorbid personality disorders influence paranoid schizophrenia. They proved urban birth, single status and avoidant personality traits are observed in young patients. Differences were more significant in male group. [60]. Detailed analyses of premorbid personality disorders and familial burden is needed to introduce more detailed models.

We proved clinical and demographical characteristics of the sample are predictors of symptom dimensions of schizophrenia and bipolar disorder. We also saw relation between clinical dimensions and course of disorder and impairment during disorder.
